# Melanin presence inhibits melanoma cell spread in mice in a unique mechanical fashion

**DOI:** 10.1038/s41598-019-45643-9

**Published:** 2019-06-26

**Authors:** Michal Sarna, Martyna Krzykawska-Serda, Monika Jakubowska, Andrzej Zadlo, Krystyna Urbanska

**Affiliations:** Department of Biophysics, Faculty of Biochemistry, Biophysics and Biotechnology, Jagiellonian University, Gronostajowa 7, 30-387 Krakow, Poland

**Keywords:** Cancer models, Cell invasion

## Abstract

Melanoma is a highly aggressive cancer that exhibits metastasis to various critical organs. Unlike any other cancer cells, melanoma cells can synthesize melanin in large amounts, becoming heavily pigmented. Until now the role of melanin in melanoma, particularly the effect of melanin presence on the abilities of melanoma cells to spread and metastasize remains unknown. Recently, we have shown that melanin dramatically modified elastic properties of melanoma cells and inhibited the cells invasive abilities *in vitro*. Here, we inoculated human melanoma cells with different melanin content into nude mice and tested the hypothesis that cell elasticity is an important property of cancer cells for their efficient spread *in vivo*. The obtained results clearly showed that cells containing melanin were less capable to spread in mice than cells without the pigment. Our findings indicate that the presence of melanin inhibits melanoma metastasis, emphasizing possible clinical implications of such an inhibitory effect.

## Introduction

Melanoma is a malignant tumor originating from transformed melanocytes – cells that normally produce melanin^1^. However, unlike in melanocytes, in which melanin synthesis is regulated by different factors and plays a specific biological role^2^, melanin pigmentation in melanoma is highly deregulated^3^. For years the role of melanin in melanoma, particularly the effect of melanin pigmentation on the metastatic behavior of melanoma cells was under extensive scrutiny with the outcomes so far being inconclusive^4^. While it is generally accepted that during melanin synthesis melanoma cells become less aggressive, hence production of melanin requires involvement of certain genes that suppress invasion^5,6^, it is what happens after the pigment is produced that remains controversial. According to some researchers pigmentation is a one-way street that determines the fate of a cell^7,8^, whereas others point to possible phenotypic switching of melanoma cells that affects metastasis^9,10^. Regardless of the view, it should be realized that melanin synthesis is only a brief episode in the entire life span of a cell lasting for hours to days at maximum. Consequently, after melanin synthesis a cell remains pigmented for many days or even weeks depending on its proliferation activity. Importantly, melanoma cells do not excrete the pigment like their normal counterparts what can result in heavy pigmentation of the cells^11^. Even though pigmentation appears to be an important factor for melanoma metastasis, the impact of melanin presence on melanoma cell behavior was never properly addressed. This seems rather surprising in light of current knowledge in the field of cancer biomechanics^12^.

Recently, nanomechanical properties of cancer cells, in particular their elasticity, have been identified as one of key parameters of the cells for their efficient spread during metastasis^13–16^. Although it was postulated long ago that soft and highly deformable cancer cells should have a facilitated ability to spread^17,18^, the importance of cell elasticity in cancer metastasis has been demonstrated in recent *in vitro*- and *in vivo*-based studies^19–21^. Thus, elasticity is viewed as an important indicator of the metastatic phenotype of cancer cells and its even been proposed as a potential diagnostic marker^22^.

In pigmented cells, including melanoma cells, melanin is in the form of distinct micro-size granules called melanosomes^23^. These organelles exhibit unusual physicochemical characteristics^24,25^ and unique nanomechanical properties^26^. Intriguingly, the impact of melanin presence on elasticity of melanoma cells has long been overlooked, despite the fact that the pigment granules were found to be exceptionally stiff and very hard to deform^27^. Consequently, the potential mechanical effect of melanin presence on the metastatic behavior of melanoma cells was never taken into consideration.

In our recent studies we have demonstrated that the presence of melanin dramatically modified nanomechanical properties of melanoma cells, significantly increasing elastic modulus of the cells^28,29^. Moreover, we have also shown that the pigment granules inhibited the invasive abilities of melanoma cells *in vitro* in a number of granule dependent manner^30^. The presence of melanin in melanoma cells did not affect any of the cell vital functions important for metastasis, such as: expression of prometastatic markers, proliferation, migration, and excretion of metalloproteinases, indicating that the inhibitory effect of melanin was solely mechanical in nature. Such observation prompted us to examine whether similar inhibitory effect of melanin presence on melanoma cell spread could be observed *in vivo*.

To address this issue we inoculated human melanoma (SKMEL-188) cells with different melanin content into nude mice and tested the hypothesis that cell elasticity is an important property of cancer cells for their efficient spread *in vivo*. Results of our study clearly showed that cells containing melanin that had high elastic modulus, were less capable to spread in mice than cells without the pigment i.e. cells with low elastic modulus. These findings shed new light on the role of melanin in melanoma metastasis and point to potential clinical implications of such inhibitory effect of the pigment.

## Results

The cells used in this study were divided into three experimental groups based on their melanin content and nanomechanical properties. First consisted of non-pigmented melanoma cells that had the lowest elastic modulus, while the remaining two consisted of pigmented melanoma cells with two different levels of melanin pigmentation: moderately-pigmented and heavily-pigmented cells that had medium and high elastic modulus, respectively. As demonstrated in our previous studies such cell model allowed us to obtain cells with different nanomechanical properties^28^, while maintaining other key cell parameters important for metastasis not altered^30^. We then inoculated the cells into immunodeficient mice to examine the impact of melanin presence on the abilities of melanoma cells to spread *in vivo*.

**Figure 1:**
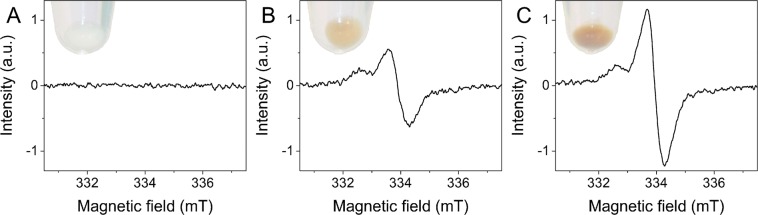
Determination of melanin in cell samples used in the experiments. EPR spectra of non-pigmented melanoma cells (**A**) and of pigmented melanoma cells with different levels of melanin pigmentation: moderately-pigmented (**B**) and heavily-pigmented (**C**) cells. The melanin EPR signal of pigmented cells is a superposition of pheomelanin signal (low field component) and eumelanin signal (high field component). Insets show images of cell pallets taken before freezing for EPR analysis. Note that the pellet of non-pigmented cells is white i.e. the cells are amelanotic, whereas the pellets of pigmented cells have a yellow-reddish color, which is typical for samples containing a mixture of pheomelanin and eumelanin.

Figure 1 shows electron paramagnetic resonance (EPR) spectra of the cells used in this study. As evident from the data non-pigmented melanoma cells did not contain any melanin i.e. no EPR signal was detected for this sample (Fig. 1A), whereas pigmented melanoma cells (Fig. 1B,C) showed distinct EPR signals with magnetic parameters characteristic for melanin free radicals^31^. Based on EPR analysis the average number of melanin granules per cell (mean ± s.d.) for each cell sample was determined and these results are shown in Table 1.

**Table 1:** Numerical values of the obtained results.

Melanoma cells	Number of melanosomes per cell^a^	Young’s modulus (kPa)^b^	Elastic deformation (µm)^c^	Number of metastatic tumors^d^	Liver mass (g)^e^
Non-pigmented	—	0.98 ± 0.41	1.32 ± 0.31	83 ± 39	2.37 ± 0.63
Moderately-pigmented	32 ± 3*	3.08 ± 1.42*	1.08 ± 0.21*	57 ± 41*	2.06 ± 0.78*
Heavily-pigmented	58 ± 4**	4.93 ± 2.38**	0.89 ± 0.14**	11 ± 8**	1.49 ± 0.09**

^a^Average number (mean ± s.d.) of melanin granules determined by EPR analysis; ^b^Average value (mean ± s.d.) of the Young’s modulus determined by AFM measurements; ^c^Average value (mean ± s.d.) of elastic deformation determined based on mechanical analysis; ^d^Average number (mean ± s.d.) of metastatic tumors found in the livers of mice during autopsy; ^e^Average value (mean ± s.d.) of liver masses determined during autopsy. *Statistically significant vs. non-pigmented cells; **statistically significant vs. moderately-pigmented cells. For all values *P* < 0.0001.

**Figure 2:**
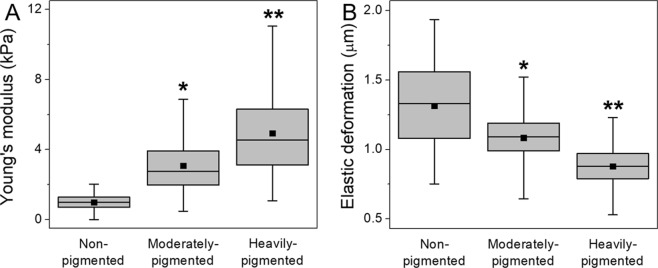
Nanomechanical properties of melanoma cells used in this study. Box plots of the Young’s modulus values (**A**) and of maximum elastic deformation (**B**) for melanoma cells with different levels of melanin pigmentation and for non-pigmented melanoma cells. Square dots in box plots indicate the median values, whereas horizontal lines represent means. *Statistically significant vs. non-pigmented cells; **statistically significant vs. moderately-pigmented cells. For all values *P* < 0.0001.

Nanomechanical properties of the cells are characterized in Fig. 2. Figure 2A shows box plot of the Young’s modulus, whereas Fig. 2B shows box plot of maximum elastic deformation. Average values (mean ± s.d.) of both the Young’s modulus and maximum elastic deformation are given in Table 1. These results indicate that non-pigmented melanoma cells had the lowest value of the Young’s modulus and exhibited highest elastic deformation. On the other hand, pigmented melanoma cells had significantly higher values of the Young’s modulus and exhibited much lower elastic deformations with the differences being more prominent in the case of heavily-pigmented cells.

**Figure 3:**
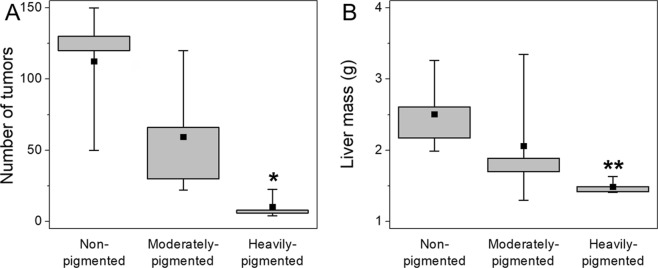
Results obtained from *in vivo* experiments. Box plots of liver masses (**A**) taken from mice, which had inoculated melanoma cells with different levels of melanin pigmentation followed by box plot of the number of metastatic tumors (**B**) that were visible on the livers during autopsy. Square dots in box plots indicate the median values. *Statistically significant vs. non-pigmented cells (*P* < 0.05); **statistically significant vs. non-pigmented cells (*P* < 0.01).

**Figure 4:**
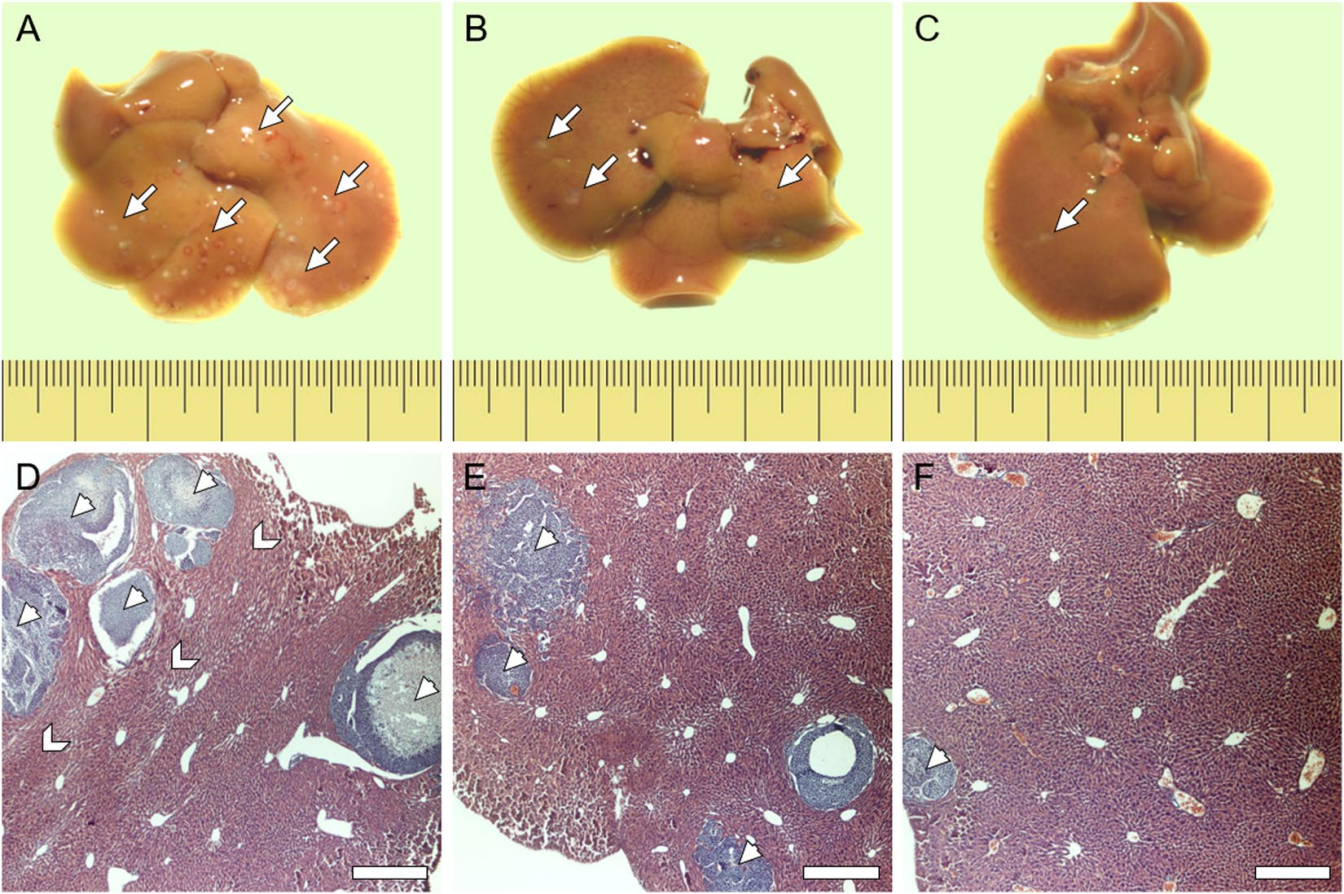
Tissue samples with metastatic tumors isolated from mice. Liver samples isolated from mice, which had inoculated melanoma cells with different levels of melanin pigmentation: non-pigmented (**A**,**D**), moderately-pigmented (**B**,**E**) and heavily-pigmented (**C**,**F**). Upper row images show entire livers, whereas lower row images show histological samples of the livers. Arrows in the upper row images indicate the locations of metastatic colonies, whereas arrow heads in the lower row images indicate individual tumors. Note the morphology of the lobules of livers in the case of mice inoculated with non-pigmented melanoma cells is much more compact than in the case of mice inoculated with pigmented cells, indicating higher tissue density. Higher tissue compaction is indicated by chevrons. Scale pitch on the rulers represents one millimeter, whereas scale bars represent 200 μm.

Results of *in vivo* experiments are shown in Fig. 3. As evident from the data, mice inoculated with non-pigmented melanoma cells developed highest number of metastatic tumors when compared to mice inoculated with pigmented melanoma cells (Fig. 3A). Moreover, in the case of mice inoculated with pigmented melanoma cells, more metastatic tumors were observed in mice, that were inoculated with moderately-pigmented cells than those with heavily-pigmented cells. Images of livers isolated from mice during autopsy (Fig. 4A–C) clearly show that mice inoculated with non-pigmented melanoma cells, contained significantly more metastatic colonies than mice inoculated with pigmented melanoma cells. Comparison of the animal livers (Fig. 3B) indicated that livers isolated from mice that were inoculated with non-pigmented melanoma cells were much heavier than livers from mice inoculated with pigmented melanoma cells. This was due to the fact that the former contained significantly more metastatic tumors than the latter. Average values (mean ± s.d.) of both the number of metastatic tumors found in the livers of mice and liver masses are shown in Table 1.

**Figure 5:**
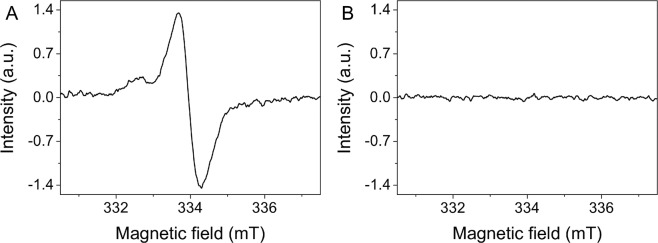
Loss of melanin in dividing melanoma cells. EPR spectra of 10^6^ heavily-pigmented SKMEL-188 cells immediately after melanin synthesis (**A**), and after 14 days of culture (**B**) under such conditions the cells did not synthesize melanin. As evident, no detectable melanin signal was observed in the cells after two weeks of culture indicating the loss of pigment by the cells due to consecutive cell division.

Microscopy analysis of histological liver samples (Fig. 4D–F) revealed that tumors formed by non-pigmented melanoma cells were larger than tumors formed by pigmented melanoma cells. Importantly, these images also showed that the metastatic tumors lack any visible melanin even in the case of tumors formed in livers of mice inoculated with heavily-pigmented cells. To confirm this observation, EPR analysis of the so called ‘zinc-enhancing effect’ for melanin detection was performed on liver samples. Supplementary Figure S1 shows results of such analysis. As evident, no enhancement of the EPR signal was observed, confirming that no measurable melanin was present in the livers containing metastatic tumors. This indicates that SKMEL-188 cells did not synthesize melanin *in vivo*. It is a key observation of this study, validating the results obtained from the mice model. It should be emphasized that any spontaneous pigmentation of the cells in mice would interfere with the observations and could lead to erroneous conclusions. Importantly, unlike melanocytes that transfer the synthesized pigment to neighboring keratinocytes, quickly reducing their melanin content, melanoma cells do not excrete the pigment^4^. The only way for melanoma cells to get rid of melanin is by dilution due to consecutive cell division. Under *in vitro* conditions, it takes approximately 10 days for heavily-pigmented SKMEL-188 cells to lose most of the pigment as a result of dilution. As determined in our previous study, the doubling time of heavily-pigmented SKMEL-188 cells was approximately 20 hours^30^. Therefore, after 14 days, the amount of cellular melanin should be reduced by about five orders of magnitude compared to the original melanin content, which would be too little to detect by EPR. Indeed, we demonstrated that no melanin could be detected in heavily-pigmented SKMEL-188 cells after 14 days of culture (Fig. 5). There is no doubt that cell proliferation *in vitro* does not reassemble that *in vivo;* however, once the cells reached a metastatic site and entered the exponential growth phase, they quickly lost the pigment. This explains why metastatic tumors found in the livers of mice did not contain melanin.

The fact that the cells lost their original pigment so quickly, is also the reason why in our study we decided to inoculate the cells into the tail vein of mice instead of a subcutaneous injection of the cells under the skin. It should be emphasized that in the latter case, the time needed for the tumors to reach a stage at which the cells would begin to metastasize would be significantly greater, resulting in many more cell divisions and further dilution of the pigment. Taking into consideration that no melanin was detected in the cells after 14 days of inoculation, this would leave the cells non-pigmented long before they started to spread. It should be emphasized that in our experimental model, based on the inoculation of melanoma cells directly into the bloodstream, the cells could start to divide once they reached a suitable niche such as a metastatic site. This indicates that the cells were pigmented during all key stages of metastasis, such as: extravasation from the blood vessels, invasion through multiple basement membranes and migration inside the tissue. Importantly, it is during all these stages of metastasis that cell elasticity plays a critical role. Hence the cells must undergo extensive deformation in order to penetrate these barriers^32^. As demonstrated in our previous work, presence of melanin in melanoma cells limited their abilities to undergo extensive deformation when passing through a mechanical barrier such as the endothelial wall or the basement membrane^30^. Therefore, we believe that melanin is responsible for the reduced abilities of pigmented melanoma cells to spread in mice.

**Figure 6:**
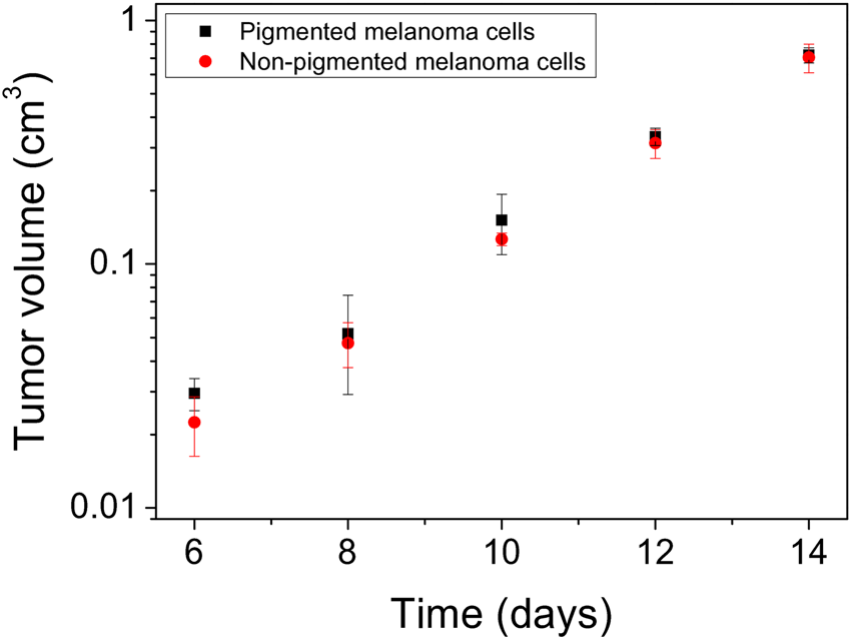
Growth curves of tumor progression for pigmented and non-pigmented SKMEL-188 cells in nude mice. Tumor volume (y axis) is plotted in logarithmic scale. To determine the volume of the tumors, the oblate spheroid approximation was used. In calculation of the spheroids volume, the two horizontal axes were averaged giving the equatorial diameter of a spheroid, whereas tumor height indicated half of the spheroid’s polar diameter.

Finally, to determine whether the larger sizes of metastatic tumors formed by non-pigmented melanoma cells were related to different proliferation abilities of the cells in mice, a comparison of the growth rates of pigmented and non-pigmented SKMEL-188 cells *in vivo* was performed. Figure 6 shows growth curves of tumor formation by pigmented and non-pigmented SKMEL-188 cells in nude mice. The data show no significant difference in the proliferation rates between pigmented and non-pigmented SKMEL-188 cells *in vivo*. Based on these results we can conclude that significantly more non-pigmented melanoma cells were present in the livers of mice at a given time, which gave rise to more metastatic colonies, and that non-pigmented cells reached the livers sooner than pigmented cells, hence the observed larger sizes of the metastatic tumors. This further confirms that highly deformable, non-pigmented melanoma cells had an increased ability to spread in mice.

Although the volume of the tumors formed by pigmented and non-pigmented SKMEL-188 cells on the back of mice was similar, the shape of the tumors was noticeably different. In the case of tumors formed by pigmented cells their shape was ‘tight’, whereas in the case of tumors formed by non-pigmented cells, their shape was rather ‘loose’ (Supplementary Figure S2). Interestingly, similar observation was made by Urbanska and others after implementation of tumor scraps containing either pigmented or non-pigmented Bomirski Hamster Melanoma cells into rodents’ eyes^33^. Tumors formed by melanotic cells were well defined, with sharp edges, whereas tumors formed by amelanotic cells were less compact having irregular borders. This could be due to different spreading capabilities of pigmented and non-pigmented melanoma cells with the former being less capable to spread than the latter. Of course, it cannot be ruled out, that the observed differences were also related to slightly different injection of the cells into the mice (e.g. angle of punctuation, depth of needle penetration, etc.). However, this factor seems unlikely considering reproducibility of the observed effect in repeated experiments.

## Discussion

In this work we examined experimentally the role of melanin pigmentation in melanoma metastasis, particularly the effect of melanin presence on the abilities of melanoma cells to spread *in vivo*. To achieve our main goal we tested the hypothesis whether cell elasticity is an important property of cancer cells for their efficient spread during metastasis. The obtained results clearly show that presence of melanin inhibits the abilities of melanoma cells to spread *in vivo* and that the effect is mostly mechanical in nature. Considering the extent of the effect of melanin on the elastic properties of melanoma cells, particularly on their deformation capabilities, we postulate that in melanoma cells melanin can be viewed as a mechanical inhibitor of the cells metastatic abilities. Of course, other cell parameters, such as the effect of cancer cells on their microenvironment^34^, connexin-formed gap junctions^35^ and cell migration^36^, among others, also play an important role in the process of metastasis of melanoma cells. Nevertheless, our findings point to a novel, mechanical role of melanin in melanoma metastasis. This is an important observation for melanoma research, considering that the potential role of melanin in melanoma metastasis was long a matter of intense debate. The controversy is mostly due to contradictory findings of different studies and lack of clinical data that would support observations gathered from basic research. However, it should be stressed that recently published clinical data on survival of patients with diagnosed melanoma point to a possible relationship between the degree of melanin pigmentation and melanoma aggressiveness^37^. In the study performed by Thomas and others, the authors found that amelanotic melanoma was associated with poorer patient survival than pigmented melanoma. Based on the clinical data, researchers have concluded that amelanotic melanoma, was more difficult to diagnose than pigmented melanoma and therefore it remained undetected longer until it exhibited a much more advanced stage. However, such explanation leaves many questions unanswered, especially those related to the mechanism of the observed effect, in particular, which of the cell parameters are responsible for such melanoma behavior. It cannot be ruled out that amelanotic melanoma spread more than pigmented melanoma and therefore at the time of diagnosis it was already at a much more advanced stage. We believe that the results of our study on the inhibiting role of melanin on melanoma cell spread may be one of the main reasons why amelanotic melanoma seems to be more aggressive in clinics than pigmented melanoma. Of course, additional experiments are required to unambiguously determine the role of melanin pigmentation in melanoma metastasis. Ideally, live tracking of cellular movement of pigmented and non-pigmented melanoma cells *in vivo* would be of particular interest. However, the inhibitory effect of melanin on melanoma metastasis, if confirmed in future studies, may have potential clinical implications for melanoma diagnosis. Quantitative analysis of melanin content in invading melanoma cells identified during histopathological analysis could facilitate simple and accurate determination of the cells metastatic potential. Cells containing more melanin would likely be less aggressive than cells without the pigment. Such analysis could better assess the risk of developing metastatic tumors, leading to a more complete diagnosis, and therefore should help in employing an optimized treatment.

## Methods

All methods described in this work were performed in accordance with relevant guidelines and regulations

### Cells

Human melanoma SKMEL-188 cells were chosen for this study for the ability of the cells to synthesize melanin under *in vitro* conditions in a relatively controlled manner. The cells were originally kept in Ham’s F10 culture medium supplemented with 10% FBS at 37 °C in a 5% CO_2_ humidified atmosphere. Under such conditions the cells did not synthesize melanin and were used as control, non-pigmented cells. For melanin synthesis, the culture medium was replaced with Dulbecco’s Modified Eagle Medium, which induces pigmentation of the cells. Different levels of melanin content in the cells were obtained as described previously^28^. After the protocol, cells were passaged and maintained in Ham’s F10 culture medium for 3 days before the experiments. This minimized any risk of shock to the cells caused by the exchange of culture media and ensured that no melanin synthesis took place during the experiments.

### Analysis of melanin in cells and tissue samples

To determine the amount of melanin in cells and tissue samples EPR analysis was performed^38^. This non-invasive technique allows quantitative determination of melanin in various samples, including cells, and can be successfully utilized to distinguish the two main types of melanin pigment: the brown-black eumelanin and the yellow-reddish pheomelanin^31^. For the analysis, cells were detached from the culture dishes, centrifuged, counted, frozen, and stored at 77 K. The number of cells for each cell sample was 10^6^. Tissue samples obtained during autopsy were snap-frozen in liquid nitrogen and kept at 77 K until the analysis. The enhanced specificity of EPR determination of melanin was obtained by examining the so-called ‘zinc-enhancing effect’^39^. In the presence of adequate concentration of zinc ions at neutral or slightly acidic pH, chelate complexes between melanin ortho-semiquinones (or ortho-semiquinonimines) and metal ions are formed, stabilizing the melanin free radicals. As a result, a shift in the equilibrium between fully reduced and fully oxidized melanin subunits, and their semi-reduced (semi-oxidized) forms occurs, which significantly increases the intensity of the observable EPR signal. The zinc-enhancing effect is very specific for melanin characterization and only samples containing natural or synthetic melanin will exhibit such an effect. For the analysis of tissue samples, liver fragments containing metastatic tumors were homogenized using TissueLyser Bead homogenizer (Qiagen). Half of the sample was suspended in PBS, whereas the remaining half was saturated with zinc acetate. In the case of cells, incubation of the samples with zinc acetate was made before freezing. Final concentration of zinc ions in the samples was 50 mM. As a standard for melanin determination, synthetic melanin, derived from enzymatic oxidation of cysteine-L-dopa, at a concentration of 0.73 mg/ml was used. EPR measurements were conducted using a Bruker EMX-AA spectrometer operating at X-band with 100 kHz magnetic modulation. Measurements were performed in liquid nitrogen using a standard finger-type quartz dewar. Detailed description of EPR analysis used in this work can be found elsewhere^40^. Average number of melanin granules per cell in the cell samples was determined as described previously^30^.

### Elasticity measurements

Nanomechanical analysis of the cells was conducted using atomic force microscopy (AFM) technique. Measurements were performed with a BioScope Catalyst atomic force microscope (Bruker) coupled with an Axio Observer Z1 inverted optical microscope from Zeiss. During the analysis cells were maintained in Ham’s F10 culture medium at 37 °C. Before measuring a cell, the AFM probe was positioned on top of a cell and aligned at the cell center using optical microscopy live view. Once aligned, force curves from a grid of 5 × 5 points were collected at a rate of 1 Hz. 50 cells for each condition were analyzed. Detailed description of the mechanical analysis used in this work can be found elsewhere^41^. To examine the effect of melanin on the nanomechanical properties of melanoma cells at the cellular level, probes with spheres attached to the end of the cantilevers were chosen. Such approach seems more appropriate when considering cell spread *in vivo* rather than classical analysis with a sharp tip that examines sub-cellular effects. The probes used in the analysis were CP-CONT-BSG-type probes from NanoAndMore with a nominal spring constant of 0.2 N/m and a sphere diameter of 10 µm. For precise calculation of the Young’s modulus values spring constants of the used cantilevers were routinely checked using the thermal tune procedure^42^. In addition to the Young’s modulus, maximum elastic deformation was determined. This is a quantity taken from a force-indentation curve that represents a point at which the experimental data starts to deviate from the elastic model. It is seems very unlikely that cancer cells could undergo non-elastic deformations, hence to obtain such deformations significant forces are required. Taking into consideration that cells are capable of generating much lower forces^43^ we assumed that the maximum elastic deformation is kind of a limit of the cells deformation capabilities *in vivo*. Supplementary Figure S3 shows example force curves of pigmented and non-pigmented melanoma cells with marked points referring to the maximum elastic deformation. Detailed description of force curve analysis used in this study, in particular, determination of the maximum elastic deformation can be found in the following work^44^.

### Animals

Analysis of melanoma cell spread *in vivo* was performed on BALB/c nude mice in compliance with the national and European regulations on animal use and was approved by the First Local Ethical Committee on Animal Testing at the Jagiellonian University in Krakow (permission no. 137/2013 obtained on October 23^rd^, 2013). Before the experiments, mice were immunized with human antigen by a subcutaneous injection of human lung adenocarcinoma A549 cells into the rear thigh. This allowed us to minimize the number of mice needed for the experiments, hence in immunodeficient mice such as BALB/c previous immunization leads to higher acceptance of human cells^45^. Time between immunization and melanoma inoculation was three months. After immunization all mice were carefully inspected for any tumor formation and only tumor-free mice were used in the experiments. For the analysis of melanoma cell spread, 10^4^ cells in a volume of 100 μl of PBS were inoculated into the tail vein of a mouse using a sterile surgical syringe. Twelve mice (four per each experimental group) were used in the analysis. To compare the growth rates between pigmented and non-pigmented melanoma cells *in vivo*, 10^6^ heavily-pigmented or non-pigmented SKMEL-188 cells were subcutaneously injected into the back of additional mice (three per each experimental group). The condition of mice was carefully monitored for the duration of the experiments. Mice were checked on a daily basis, whereas the size of the growing tumors on the back of mice was determined every other day using a caliper.

### Autopsy and analysis of tissue samples

All animals were euthanized 14 days after melanoma cell inoculation. During autopsy each mouse was carefully examined for the presence of metastatic tumors. The following organs were inspected: brain, heart, skin, liver, lungs, spleen, peritoneum, intestines, stomach, bladder, and kidneys. All organs were weighted and checked by three independent researchers. Apart from few tumors found in the skin and heart of individual mice, vast majority of the metastatic tumors were located in the livers and these organs were used for quantitative analysis.

### Histology

Tissue samples obtained during autopsy were fixed in 5% buffered formalin (pH 7.4), embedded in paraffin and histologically sectioned into 5 µm thick slices using a Shandon Finesse 325 Microtome from Thermo. Standard hematoxylin and eosin staining was performed as described elsewhere^46^. Images of tissue samples were obtained using a Nikon Eclipse Ti-U inverted optical microscope coupled with a colored camera under a Plan 4 × objective. Image analysis was made using NIS-Elements F 3.0 software.

### Statistical analysis

Statistical significance of difference between mean values for AFM and EPR data was determined using a two-sample independent Student’s t-test at the 95% confidence level. For the analysis of data obtained from *in vivo* experiments a Single Factor ANOVA test was employed. Both mean and median values were reported as appropriate. Differences among means were reported using approximated *P* values. Statistical analysis was made using the Mathematica 8.0 software.

## Supplementary information

Supplementary Information
