# Supplementary material for: Melanin presence inhibits melanoma cell spread in mice in a unique mechanical fashion
Source: Sci Rep. 2019 Jun 26;9:9280. doi: 10.1038/s41598-019-45643-9 (PMC6594928; doi:10.1038/s41598-019-45643-9)
Supplement: Supplementary file 1 — Supplementary Information [file 41598_2019_45643_MOESM1_ESM.pdf]

Supplementary Information For:

Melanin presence inhibits melanoma cell spread in mice in a unique mechanical fashion

Michal Sarna*, Martyna Krzykawska-Serda, Monika Jakubowska, Andrzej Zadlo, Krystyna Urbanska

Department of Biophysics, Faculty of Biochemistry, Biophysics and Biotechnology, Jagiellonian University, Gronostajowa 7, 30-387 Krakow, Poland

*Corresponding author: Michal Sarna, PhD

E-mail: michal.sarna@uj.edu.pl

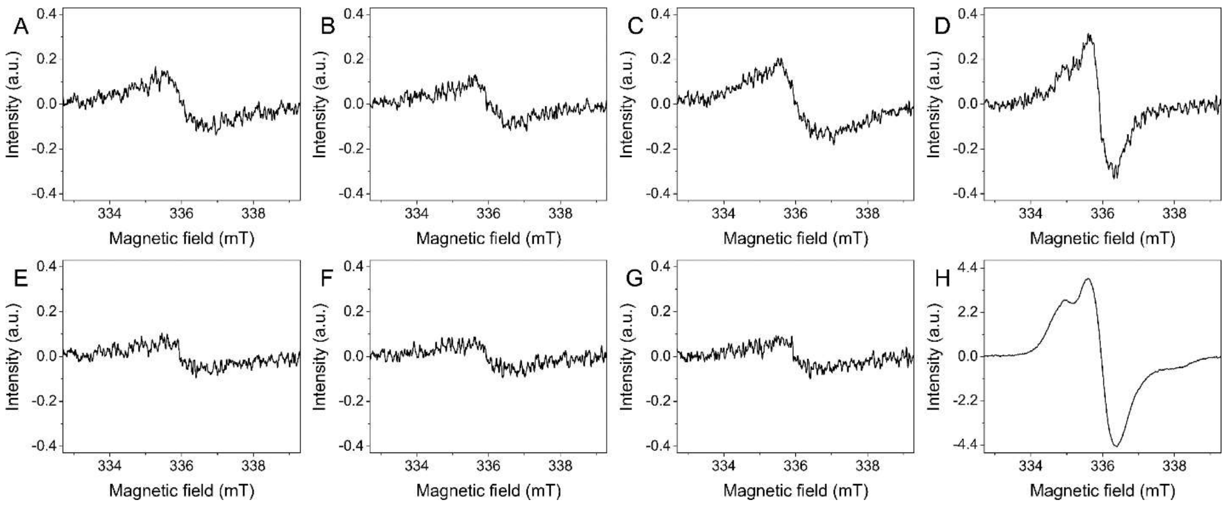

Figure S1. Analysis of the zinc-enhancing effect. EPR spectra of liver samples isolated from mice inoculated with melanoma cells that contained different amounts of melanin: non-pigmented cells (**A, E**), moderately-pigmented cells (**B, F**), heavily-pigmented cells (**C, G**) and of cysteine-L-dopa melanin (**D, H**) used as melanin standard. Upper row images show EPR spectra of the samples in PBS, whereas lower row images show EPR spectra of the same samples saturated with zinc acetate run under identical conditions. As evident, only sample containing melanin exhibits a significant zinc-dependent increase of the EPR signal, whereas no zinc effect is observed in the case of liver samples, excluding the presence of melanin in these samples. The origin of the detected signal in the livers is clearly non-melanotic.

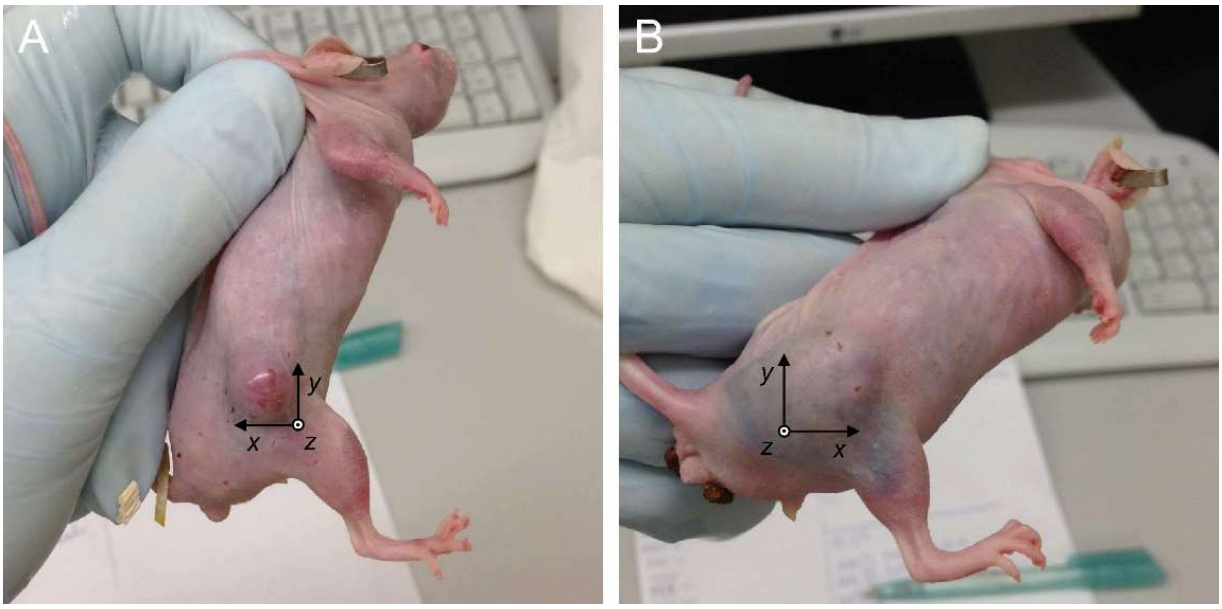

Figure S2. Comparison of melanoma tumors formed by pigmented and non-pigmented SKMEL-188 cells in nude mice. Images show melanoma tumors formed by heavily-pigmented (**A**) and non-pigmented (**B**) SKMEL-188 cells at 14th day after subcutaneous injection. Note that tumors formed by injection of pigmented cells are more 'bulged', whereas tumors formed by non-pigmented cells are more 'flattened'.

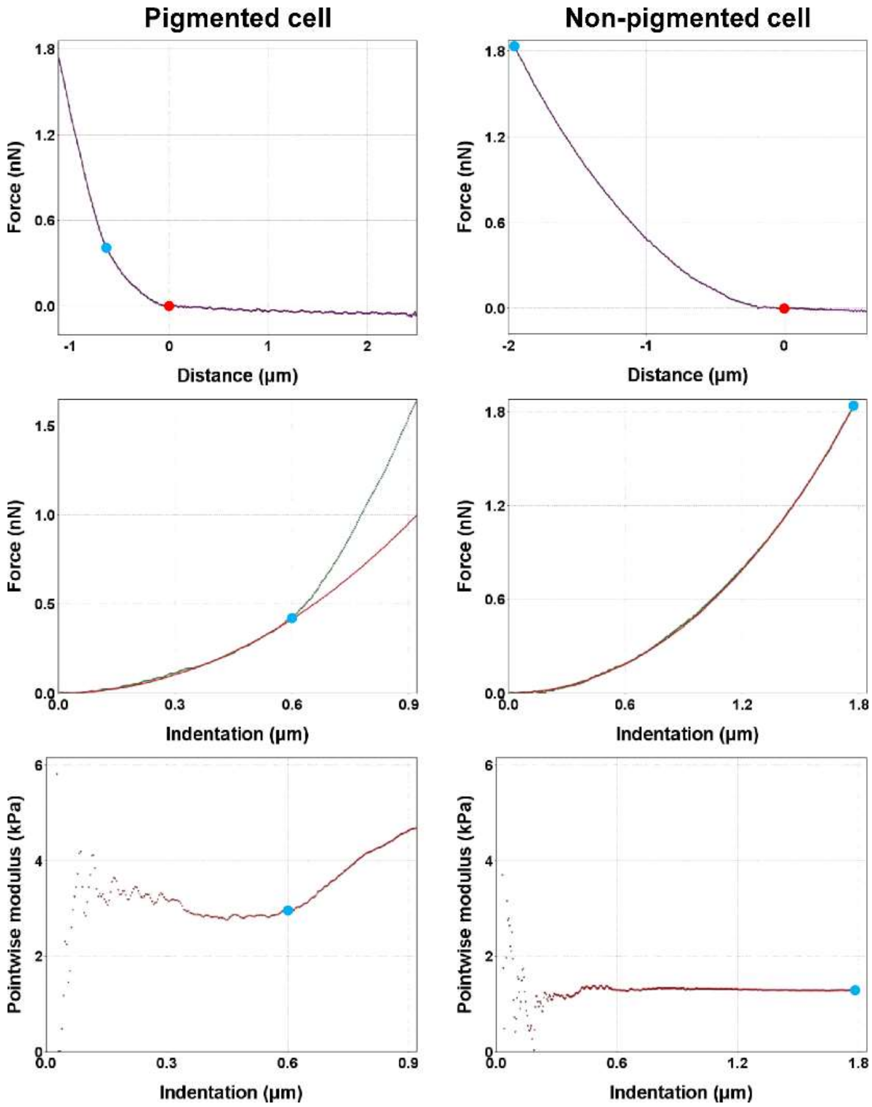

Figure S3. Example force curves of the cells analyzed in this work. Force-distance curves (upper row) for pigmented and non-pigmented melanoma cells with indicated: point of contact (marked with red dot) and point of maximum elastic deformation (marked with blue dot). Force-indentation curves (middle row) with fit of the Hertz model (solid line) clearly show the transition point in the case of a pigmented cell from which the experimental data do not follow the elastic model. The same effect can also be seen on the pointwise modulus data (bottom row). Pointwise modulus-indentation curve for a pigmented cell shows significant increase in the values of the Young's modulus starting from the transition point. It should be emphasized that such transitions were only observed in the case of pigmented cells, which indicates that presence of melanin granules in these cells not only made the cells stiffer but also modified their mechanical properties, elasticity in particular. In non-pigmented cells, on the other hand, the values of the Young's modulus did not change over the entire indentation depth and no such transitions were recorded.
